# Response of *Pseudokirchneriella subcapitata* in Free and Alginate Immobilized Cells to Heavy Metals Toxicity

**DOI:** 10.3390/molecules25122847

**Published:** 2020-06-19

**Authors:** Zaki M. Al-Hasawi, Mohammad I. Abdel-Hamid, Adel W. Almutairi, Hussein E. Touliabah

**Affiliations:** 1 Biological Sciences Department, Faculty of Science, King Abdulaziz University, P.O. 80203, Jeddah 21589, Saudi Arabia; 2 Botany Department, Faculty of Science, University of Mansoura, Mansoura 35516, Egypt; mhamid@mans.edu.eg; 3 Biological Sciences Department, Rabigh-Faculty of Science & Arts, King Abdulaziz University, P. O. box 344, Rabigh 21911, Saudi Arabia; aalmutairi@kau.edu.sa (A.W.A.); hussein_touliabah@women.asu.edu.eg (H.E.T.)

**Keywords:** Alginate, immobilizing algal, heavy metals, toxicity, *Raphidocelis subcapitata*, *Pseudokirchneriella subcapitata*

## Abstract

Effects of 12 heavy metals on growth of free and alginate-immobilized cells of the alga *Pseudokirchneriella subcapitata* were investigated. The tested metals ions include Al, As, Cd, Co, Cr, Cu, Hg, Se, Ni, Pb, Sr, and Zn. Toxicity values (EC_50_) were calculated by graphical interpolation from dose-response curves. The highest to the lowest toxic metals are in the order Cd > Co > Hg > Cu > Ni > Zn > Cr > Al > Se > As > Pb > Sr. The lowest metal concentration (mg L^−1^) inhibiting 50% (EC_50_) of algal growth of free and immobilized (values in parentheses) algal cells were, 0.018 (0.09) for Cd, 0.03 (0.06) for Co, 0.039 (0.06) for Hg, 0.048 (0.050) for Cu, 0.055 (0.3) for Ni, 0.08 (0.1) for Zn, 0.2 (0.3) for Cr, 0.75 (1.8) for Al, 1.2 (1.4) for Se, 3.0 (4.0) for As, 3.3 (5.0) for Pb, and 160 (180) for Sr. Free and immobilized cultures showed similar responses to Cu and Se. The free cells were more sensitive than the immobilized ones. Accordingly, the toxicity (EC_50_) of heavy metals derived only form immobilized algal cells might by questionable. The study suggests that batteries of alginate-immobilized algae can efficiently replace free algae for the bio-removal of heavy metals.

## 1. Introduction

Heavy metals are natural constituents of the Earth’s crust. In undisturbed natural aquatic habitats, the processes of metal release and accumulation are fairly balanced. This balance has been disturbed by human activities that result in excessive environmental heavy metal pollution. Heavy metal contaminated environments pose great risks to human health and the function, integrity, and stability of ecosystems [1]. Heavy metal contamination has led to serious health episodes (e.g., Minamata Bay and itai-itai disease, Japan) worldwide. Therefore, it is important to acquire adequate scientific knowledge about the biological effects and environmental behavior of toxic metals.

Metal contamination of aquatic environments affects organisms at the biochemical, cellular, community and population levels. As a result of the ecological importance of algae as primary producers in most aquatic food chains, both in freshwater and marine ecosystems, algae have been frequently used as test organisms to monitor environmental quality and assess heavy metal toxicity [2,3].

Algal bioassays are considered valid methods of determining water quality by determining the stimulatory and inhibitory effects of water samples on the growth of a test species [4]. Biotests using *Pseudokirchneriella subcapitata* appear to be the only type of algal bioassay in which the methodology is sufficiently reliable and accurate [5,6]. Algal toxicity tests based on growth inhibition over 72 h. have been extensively used to assess the toxicity of contaminants in natural waters [7,8,9].

The attachment of living microorganisms to each other and to solid surfaces has been the inspiration for exploring cell immobilization techniques. Immobilized eukaryotic cells, including algae, have several advantages when compared with cells in free suspension, including a reduced tendency to become senescent and a fairly constant metabolic activity over time [10,11,12]. Cell immobilization has been shown to be essential for various uses in biotechnology and experimental biology [11,13,14,15,16].

The use of immobilized algae in toxicity testing is a relatively new approach [11,17]. Immobilization techniques could expand the utility of algal toxicity assays under field conditions. Moreover, the use of immobilized cells may overcome problems associated with toxicity testing of colored solutions and turbid effluents. Immobilization of algal cells onto suitable matrices has led to the development of a variety of algal biosensors that can evaluate toxicity and environmental quality [15,16].

Alginic acid or alginate, the salt of alginic acid, is the common name given to a family of linear polysaccharides containing 1,4-linked β-d-mannuronic and α-l-guluronic acid residues arranged in a non-regular blockwise order (Haug et al., 1966). Alginate has shown high adsorption capacity towards many divalent cations including Cd^2+^, Cu^2+^, Pb^2+^, Cr^3+^, and Hg^2+^ [11,18,19,20]. The high metal biosorption capacity of some brown algae has been attributed to the properties of cell wall constituents, such as alginate and fucoidan, which are chiefly responsible for heavy metal chelation and ion exchange properties [19]. The increased use of alginate-immobilized algae for toxicity assessment and the high affinity of alginate towards divalent metals suggested that alginate-immobilized algal cells may be useful in evaluating environmental contamination by heavy metal ions. This investigation compared the sensitivity of alginate-immobilized and free cultures of the standard green chlorococcal alga *Pseudokirchneriella subcapitata* towards the toxicity of 12 heavy metals ions: Al, As, Cd, Co, Cr, Cu, Hg, Ni, Pb, Se, Sr, and Zn. Six of these tested metals (Co, Cr, Cu, Ni, Se, and Zn) are essential trace elements for some species of algae, whereas the other six (Al, As, Cd, Hg, Pb, and Sr) are considered non-essential. All 12 of these metals are well known environmental contaminants that are potentially toxic to aquatic life at relatively high concentrations.

## 2. Materials and Methods

### 2.1. Test Alga

The chlorococcal alga *Pseudokirchneriella subcapitata*, strain NIVA-CHL1, previously known as *Selenastrum capricornutum* and *Raphidocelis subcapitata* (Nygaard et al., 1986), was cultured in standard algal assay nutrient medium (AAM) [6]. The culture vessels were placed on a shaking table at 22 ± 2 °C under continuous illumination (approx. 70 µ E m^−2^ s^−1^) provided by white fluorescent tubes. Five-day-old cultures were used for the immobilization procedure and for free algal bioassays.

### 2.2. Metals

The tested metal ions were prepared from highly pure compounds (Table 1).

Stock solutions containing 3.2 g L^−1^ (Stock A) and 1.0 g L^−1^ (Stock B) of a given metal were prepared in de-ionized glass distilled water. Metals were tested immediately after preparation.

### 2.3. Alginate Source

Alginic acid sodium salt (Sigma Cat No. A-7128, Munich, Germany) was used to prepare alginate matrices.

### 2.4. Immobilization Procedure

Algal cells were immobilized as described previously by Abdel-Hamid [21]. Briefly, sodium alginate was dissolved to a concentration of 4% (w/v) in warm AAM nutrient medium. The solution was autoclaved, cooled to room temperature and mixed with an algal suspension of known cell density (cells mL^−1^). After thorough mixing, an aliquot of this mixture was transferred to a 50 mL burette and extruded drop wise into 0.03 M CaCl_2_ solution. The burette was kept full of the alginate–algae mixture to assure a constant flow rate of about 5 drops s^−1^ (5 beads s^−1^, approx. 16 beads mL^−1^ mixture). The beads were maintained in CaCl_2_ for at least 30 min to allow complete hardening of the alginate, washed several times with distilled water, transferred to flasks containing AAM medium and stored in the dark at 4 °C until use. The initial cell density per alginate bead was calculated using simple empirical equations [21].

### 2.5. Toxicity Testing

#### 2.5.1. Microplate Procedure with Free Cells

The toxicity tests were performed in polystyrene microplates containing 6 × 4 flat bottom wells, each of 3 mL capacity [22]. A volume of 5-day-old algal culture was diluted with growth medium to prepare inoculums of 10^4^ algal cells mL^−1^, and 1.8 mL of this algal inoculum was added to each well on the plate. To the first and second wells on the plate were added 0.2 mL stock A and stock B solutions of each metal, respectively. The contents of each well were thoroughly mixed, and 0.2 mL was transferred from well 1 to well 3 and from well 2 to well 4. These serial dilutions were repeated down the row of wells on two adjacent plates, yielding 11 concentrations of each metal ion (320, 100, 32, 10, 3.2, 1.0, 0.32, 0.1, 0.032, 0.01, and 0.0032 mg L^−1^), with the twelfth well being a control. To ensure equal volumes of test solutions in all microplate wells, aliquots of 0.2 mL were removed from wells 11 and 12. Each concentration was tested in quadruplicate (four wells each). The plates were incubated for 4 days under the same algal culture conditions.

#### 2.5.2. Microplate Procedure with Algae-Alginate Beads

The procedure used to test metal toxicity toward alginate-immobilized algal cells was similar to that used for free algal cells, except that two algae-alginate beads (1.01 × 10^4^ cells bead^−1^) were added to each well.

#### 2.5.3. Toxicity-Response Parameter

At the end of the test, the beads were removed from each well and dissolved in 1% sodium hexametaphosphate solution (one bead per mL). The density of the de-immobilized algae and free cultures in each well was determined using a Coulter Multisizer (Software Level 1.8). At the same time the corresponding mean cell volume (MCV, µm^3^ cell^−1^) was determined. The toxicity endpoint was algal dry weight, calculated from mean cell density (n = 4) and MCV using the Equation (1) (Steinman and Lamberti) [23].

(1) $$\mathrm{mg}\;\mathrm{dry}\;\mathrm{wt}.\;\mathrm{of}\;S{.\;\mathrm{capricornutum}\;L}^{-1}={\mathrm{cell}\;\mathrm{count}\;\mathrm{mL}}^{-1}{\;\times \;\mathrm{MCV}\;(µm}^{3}\times 2.9\times 10^{-7})$$

#### 2.5.4. Calculation of EC_50_ and EC_100_

EC_50_ is the effective median concentration of a reagent that inhibits algal growth 50%, compared with control culture. The toxicity response parameter (algal dry weight), measured as percent relative to control (control = 100%), was plotted against the logarithm of the corresponding effluent concentration. A curve was fitted to the points with a third level polynomial, allowing EC_50_ to be determined by straight-line graphic interpolation [24]. The lowest concentration of a reagent resulting in no observable algal growth (EC_100_) was visually determined.

### 2.6. Statistical Analysis of Data

The percent of variance guideline, proposed by Miller et al. [5] and modified by Abdel-Hamid et al. [25] was used to ascertain whether the differences obtained in growth (algal dry weight) of replicate microplate wells differed significantly. Outliers were rejected according to this guideline. Significant differences between EC_50_ values obtained with free and immobilized algal cultures were assessed by t-tests. Unless otherwise indicated, differences were considered significant at the 95% confidence level (*p* ≤ 0.05). All statistical analyses were performed using proper statistical software STATISTICA Ver. 8 (StatSoft, Inc., 2007, Tulsa, OK, USA).

## 3. Results and Discussion

**Figure 1:**
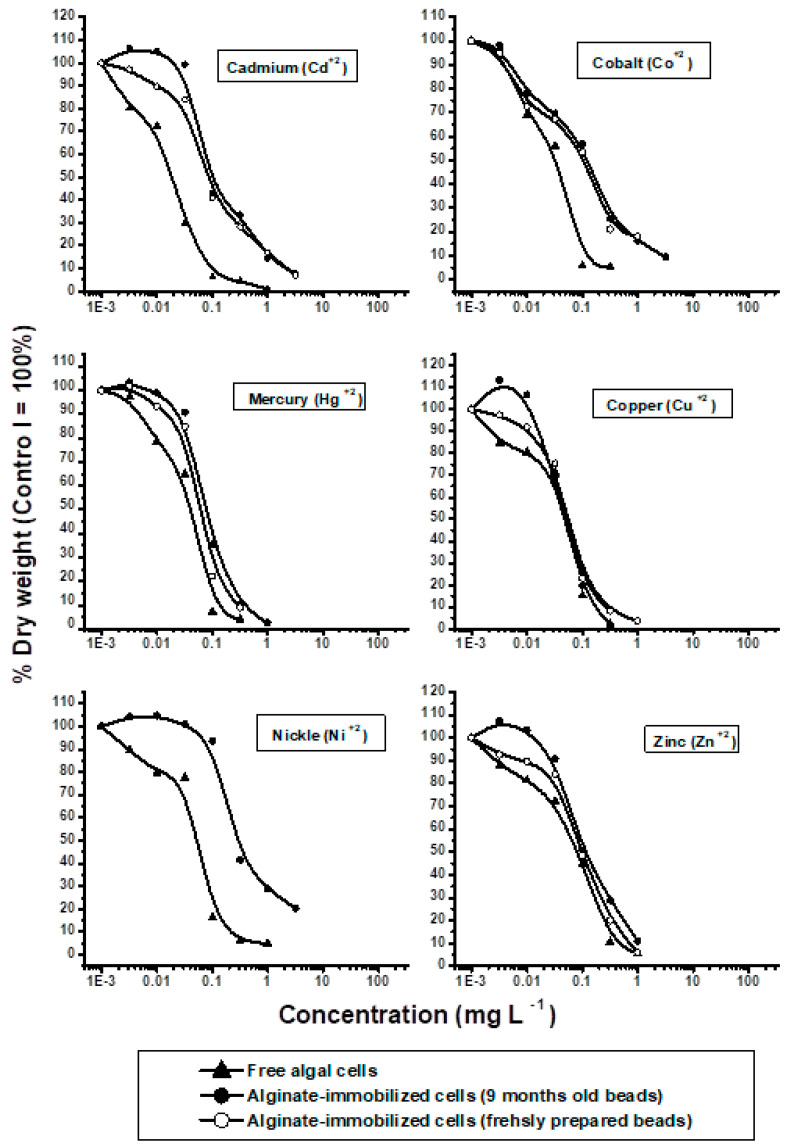
Effect of Cd, Co, Hg, Cu, Ni and Zn on growth of free and alginate- immobilized cells of *Pseudokirchnerilla* *subcapitata*.

**Figure 2:**
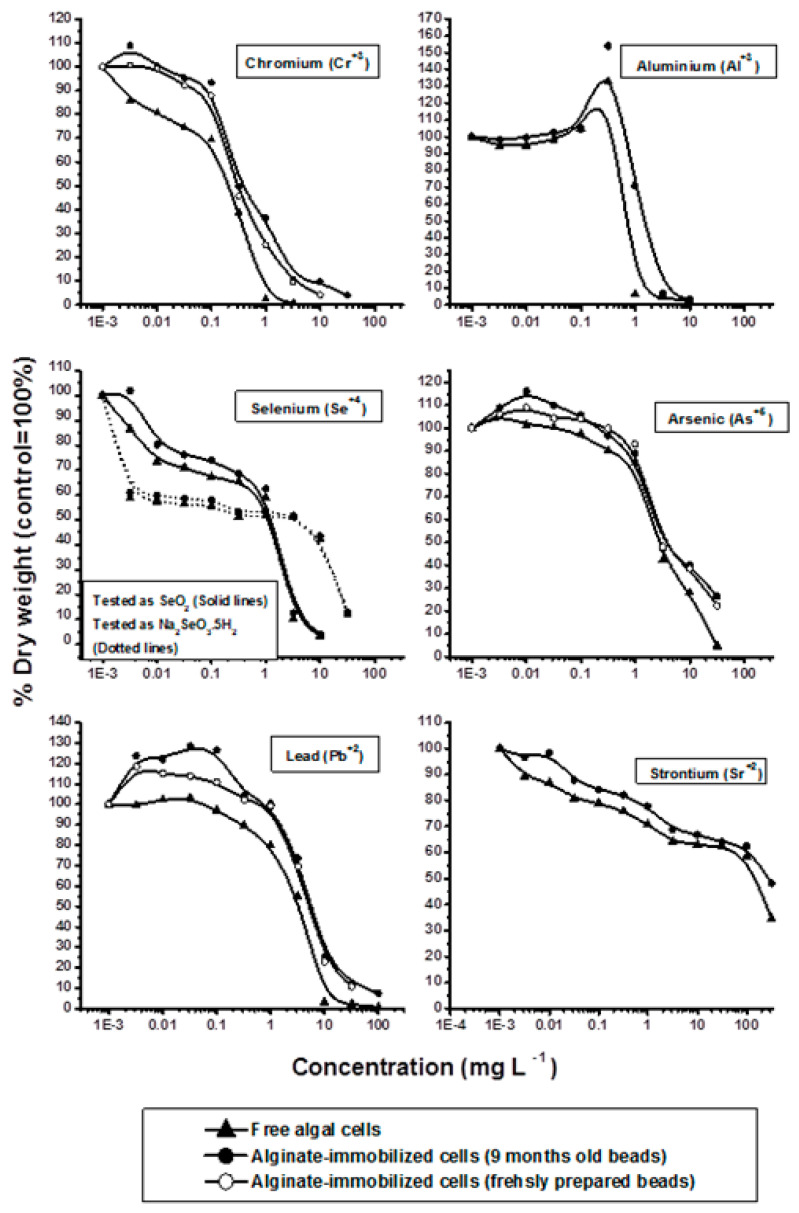
Effect of Cr, Al, Se, As, Pb and Sr on growth of free and alginate-immobilized cells of *Pseudokirchnerilla subcabitata*.

The responses of free and alginate-immobilized cells to different concentrations of metal ions are shown in Figure 1 and Figure 2. Toxicities (EC_50_ and EC_100_) calculated from dose-response curves (Figure 1 and Figure 2) are shown in Table 2. Based on EC50 values, the tested metals ranked from the most to the least toxic in the order Cd^2+^ > Co^2+^ > Hg^2+^ > Cu^2+^ > Ni^2+^ > Zn^2+^ > Cr^3+^ > Al^3+^ > Se^4+^ > As^5+^ > Pb^2+^ > Sr^2+^ (Table 2). Since metal toxicity to algae is highly pH dependent [11,26], all tests of toxicity against free and alginate-immobilized algal cells were performed at a fixed pH of 7.2.

Cadmium (Cd^2+^) inhibited the growth of both free and immobilized algal cells of freshly prepared alginate beads [27,28]. However, low cadmium concentrations slightly stimulated the growth of algal cells in stock alginate beads (Figure 1). Cadmium was significantly more toxic to free (EC_50_ = 0.081 mg L^−1^) than to immobilized (EC_50_ = 0.1 mg L^−1^) cells (*p* ≤ 0.001), with cadmium concentrations of 0.1 and 1.0 mg L^−1^ completely inhibiting the growth of free and immobilized cells, respectively (Table 2).

A study of the algistatic effects of cadmium on the growth of *P. subcapitata* showed that growth inhibition started at 20 µg L^−1^ and was complete at 80 µg L^−1^ [29]. Moreover, the EC_50_ of Cd^2+^ to *P. subcapitata* was reported to range from 20 to 50 µg L^−1^ [30]. Although the toxicity of cadmium differed slightly in the present and earlier studies, the results were generally comparable.

Cadmium is also highly toxic to other species of algae. For example, Cd^−^ inhibited the growth and reduced the chlorophyll content of *Chlamydomonas reinhardtii*, and the lethal effect on this alga at higher concentrations [31]. Cadmium ions have also been found to significantly affect the growth of *Scenedesmus obliquus* and *Euglena gracilis* [32,33]. Cadmium inhibits the synthesis of chlorophyll and carotenoids in many algal species [34].

Although usually considered a nonessential element, cadmium may be essential under certain circumstances. In zinc-depleted coastal and marine waters, for example, cadmium can play a direct role in the enzyme carbonic anhydrase in diatoms [35,36,37].

The metal ion showing the second greatest toxicity to the growth of *P. subcapitata* was Co^2+^. Even at the lowest concentrations, cobalt reduced algal growth, being significantly more toxic to free (EC_50_ = 0.03 mg L^−1^) than to immobilized (EC_50_ = 0.061 mg L^−1^) cells (*p* ≤ 0.001). Cobalt concentrations of 0.32 mg L^−1^ and 1.0 mg L^−1^ completely inhibited the growth of free and immobilized cells, respectively [28].

At low concentrations, cobalt was found to be an essential micronutrient for some microalgae [37]. Cobalt in photosynthetic organisms has been reported to catalyze essential life processes that involve carbon assimilation [38]. Cobalt has also been shown to play a crucial role in the expression and regulation of carbonic anhydrase, an enzyme essential to carbon assimilation in photosynthetic organisms [37].

Although little is known about the toxicity of Co^2+^ toward microalgae, relatively high Co^2+^ levels have been reported to significantly reduce the growth of *Nitzschia closterium* [39]. Moreover, Co^2+^ concentrations <0.01 M were found to have a toxic effect on the growth of the green alga *Chlorella pyrenoidosa* [40,41], suggesting that this early toxic effect of Co^2+^ was primarily related to its reduction of the efficiency of light reactions of photosynthesis, resulting in reduced algal productivity. The growth and pigment content of the freshwater algae *Monoraphidiu mminutum* and *Nitzschia perminuta* were found to be slightly increased at low Co^+2^ concentrations and severely inhibited at high Co^+2^ concentrations [42].

The metal showing the third highest toxicity to the growth of *P. subcapitata* was mercury, with the lowest concentrations of Hg^2+^ reducing the yield of free algal cells. However immobilized cells of both fresh and stock alginate beads showed low resistance to Hg^2+^ concentrations as high as 32 µg L^−1^, with higher concentrations inhibiting growth. Mercury was less toxic to the alginate-immobilized cells (EC_50_ = 0.056 mg L^−1^) than to free cells (EC_50_ = 0.039 mg L^−1^), with concentrations of 0.032 mg L^−1^ and 1.0 mg L^−1^ completely inhibiting the growth of free and immobilized cells, respectively [28].

Mercury is widely distributed in aquatic environments and is considered the metal most toxic to algae [3]. Mercury has been reported to inhibit algal growth partially or totally, to inhibit photosynthesis, to decrease nitrogen fixation and to reduce chlorophyll content [43,44,45,46].

Of the 12 metals tested, Cu^2+^ ranked as the fourth most toxic, with Cu^2+^ concentrations below 0.032 mg L^−1^ inhibiting the growth of free algal cells. Cu^2+^ had little effect on the growth of immobilized cells in freshly prepared beads (Figure 1), whereas Cu^2+^ concentrations below 0.032 mg L^−1^ slightly enhanced the growth of algal cells in stock 9-month-old alginate beads. Cu^2+^ concentrations above 0.032 mg L^−1^ markedly reduced the growth of both free and immobilized cells. Copper was similarly toxic to free (EC_50_ = 0.048 mg L^−1^) and immobilized (EC_50_ = 0.05 mg L^−1^) cells, with a concentration of 0.1 mg L^−1^ completely inhibiting the growth of both free and immobilized cultures [28].

Trace amounts of copper are essential for metabolic processes in algae [47]. Copper ions are directly involved in the photosynthetic electron transport chain as a plastocyanin constituent [48]. Higher Cu concentrations, however, are extremely toxic to algae. Copper inhibits growth of algae as well as photosynthesis [49]. Similar to other heavy metals, copper affects the permeability of plasma membranes, causing loss of potassium from algal cells [50,51,52]. Copper concentrations >1.0 mg L^−1^ was found to adversely affect chloroplast ultrastructure, pigment, and lipid biosynthesis, as well as being involved in the breakdown of chlorophylls and carotenoids and inhibiting PSI and PSII electron transport in *Scenedesmus* cells [53]. Copper concentrations below 1.0 mg L^−1^ significantly inhibited photosynthesis and endogenous respiration in *Chlamydomonas reinhardtii* cells [54].

The growth of free algal cells was markedly reduced by Ni^2+^, even at very low concentrations. In contrast, Ni^2+^ concentrations up to 0.032 mg L^−1^ stimulated the growth of alginate-immobilized cells. Ni^2+^ concentrations of 0.1 mg L^−1^ slightly inhibited the growth of these cells, with growth sharply declining at higher concentrations. Nickel was significantly (*p* ≤ 0.001) more toxic to free (EC_50_ = 0.055 mg L^−1)^ than to immobilized (EC_50_ = 0.3 mg L^−1^) algal cells, with the growth of these cells completely inhibited by Ni^2+^ concentrations of 0.1 mg L^−1^ and 3.2 mg L^−1^, respectively.

Compared with other heavy metals, less is known about the effects of Ni^2+^ on algae. Nickel is accumulated by algae and inhibits algal growth [55,56]. Carbon metabolism, primary Calvin cycle reactions, is the primary target of Ni^2+^, with this metal having an indirect effect on primary photochemical processes caused by disturbances of the Calvin cycle [53].

Zn^2+^ dose-dependently reduced the growth of free and immobilized cells of freshly prepared alginate beads. Although Zn^2+^ at concentrations up to 0.01 mg L^−1^ slightly enhanced the growth of algal cells in stock alginate beads, higher concentrations markedly inhibited cell growth. Zn^2+^ was slightly more toxic to the growth of free (EC_50_ = 0.08 mg L^−1^) than of immobilized (EC_50_ = 0.11 mg L^−1^) algal cells, with neither free nor immobilized cells able to grow at Zn concentrations above 0.32 mg L^−1^.

Zinc is a widely used heavy metal and is found in appreciable concentrations in aquatic habitats [57]. Zinc is an important micronutrient for the growth and metabolism of various algae [58,59]. The metabolism of zinc has been studied in many algal species, especially in *Euglena* [48]. Zn^2+^ deficient media were found to inhibit cell division in *Euglena gracilis,* indicating that Zn^2+^ is essential for pre-mitotic biochemical events, including the initiation of DNA synthesis [60]. Zn has been shown to play crucial roles in the expression and regulation of carbonic anhydrase [37] and in maintaining the integrity of ribosomes [46]. At high concentrations, Zn^2+^ was found to inhibit the growth of various algae [2,46]. High concentrations of Zn^2+^ in algal cultures reduce chlorophyll content [3] and markedly reduce photosynthesis [61].

All chromium (Cr^3+^) concentrations tested inhibited the growth of free cell cultures. Low concentrations of Cr^3+^ slightly stimulated the growth of immobilized algal cells. These cells tolerated Cr^3+^ concentrations as high as 0.1 mg L^−1^, with higher levels inducing a sharp reduction in growth. Cr^3+^was significantly (*p* ≤ 0.001) more toxic to free (EC_50_ = 0.2 mg L^−1^) than to immobilized (EC_50_ = 0.35 mg L^−1^) cells, with the growth of these cells completely inhibited by Cr^3+^concentrations of 1.0 mg L^−1^ and 3.2 mg L^−1^, respectively.

The classification of hexavalent chromium as a known human carcinogen has raised concerns about the carcinogenic potential of trivalent chromium (Glaser et al., 1986). Trace amounts of chromium are essential to algae [37]. Sublethal doses of Cr^6+^ have been reported to stimulate the nitrogen fixation activity of the cyanobacterium *Aulosira fertilissima* [62].

Similar to all other heavy metals, high chromium concentrations are toxic to algae. Cr^6+^ was shown to inhibit the proliferation of *Scenedesmus acutus* cells and their formation of coenobia (Corradi and Gorbi 1993). High concentrations of chromium inhibit protein synthesis (Rai and Dubey, 1988). Cr^6+^ was shown to dose-dependently reduce the growth of the green alga *Chlorella pyrenoidosa*, with an EC_50_ of 1.6 mg L^−1^ and an EC_100_ of 20 mg L^−1^ [41,63]. Generally, many laboratory studies have indicated that chromium is toxic to algae [64].

Free and immobilized algal cultures responded similarly to treatment with Al^3+^. Growth of both cultures remained generally unchanged at Al^3+^ concentrations up to 0.01 mg L^−1^. Slight growth stimulation was observed at an Al^3+^ concentration of 0.032 mg L^−1^ and peaked at 0.320 mg L^−1^, with the latter concentration showing 35% and 55% higher growth of free and immobilized cultures, respectively, compared with control cultures. The growth of free and immobilized cultures declined sharply at Al^3+^ concentrations of 1.0 mg L^−1^ and was completely inhibited at concentrations of 1.0 mg L^−1^ and 3.2 mg L^−1^, respectively. Free algal cells were more sensitive to Al^3+^ (EC_50_ = 0.75 mg L^−1^) than immobilized cells (EC_50_ = 1.8 mg L^−1^).

Incubation of Al^3+^ with test algae resulted in a bell-shaped growth curve, particularly at low Al^3+^ concentrations. Low Al^3+^ concentrations stimulated growth stimulation, with growth peaking at specific concentrations, and declining sharply thereafter. This finding suggests that Al^3+^ may be an essential trace element for the test algae. Other test algae responded similarly to Al^3+^ treatments (unpublished data). Although Al^3+^ is not considered a nutrient for microorganisms [65], the present findings suggest the need for additional research to determine whether Al^3+^ plays a role as a micro-essential nutrient to algae.

Increasing acidification of soil and water can result in the mobilization of aluminum in both terrestrial and limnic ecosystems (Lawrence et al., 1986). Less is known, however, about the effects of aluminum on microalgae than about its effects on fish and invertebrates. Increased aluminum concentrations are toxic to plants (Horst, 1985), as well as inhibiting the growth of the algae *Scenedesmus* sp. and *Chlorella* sp. [41,65], with the toxicity of Al to test algae being highly pH dependent.

This study tested the effects on algae of two selenium compounds, selenium dioxide (SeO_2_) and sodium selenite (Na_2_SeO_3_·5H_2_O), with Se^4+^ found to inhibit algal growth. The responses of free and alginate immobilized algal cells to Se^4+^ were comparable, and the EC_50_ values of Se^4+^ toward free and immobilized cells were identical (3.0 mg L^−1^). However, the EC_100_ values of selenium dioxide and sodium selenite were 3.2 mg L^−1^ and 32.0 mg L^−1^, respectively.

A study of the effect of selenium on the growth of six chlorococcal algae and three cyanobacteria found that, although *P. subcapitata* responded negatively to all selenium concentrations, the other algae tested showed both positive and negative responses depending on selenium concentration and compound [66]. These species-dependent variations in algal sensitivity to selenium indicate that the biotesting procedures using a single species of algae may have distinct limitations. Selenium is an essential element, acting as a cofactor in metabolic enzymes (Furness and Rainbow, 1990). Selenium was found to be a micronutrient required by some algae [67,68]. At elevated concentrations, selenium and its derivatives are considered quite toxic to life forms [69] and have become objectives of increasing environmental concern.

As^5+^ concentrations below 0.1 mg L^−1^ and 0.320 mg L^−1^ were found to slightly stimulate the growth of free and immobilized algal cells, respectively, with the growth of immobilized cells being significantly higher than that of free cells. Further increases in As^5+^ concentration resulted in marked growth decline of both free and immobilized cultures, indicating that these higher concentrations are toxic to the test algae. As^5+^ was more toxic to free (EC_50_ = 3.0 mg L^−1^) than to immobilized (EC_50_ = 4.0 mg L^−1^) algal cells, with the growth of both cultures being completely inhibited at As^5+^ concentrations of 32 mg L^−1^.

Arsenic is a highly toxic element, having various well-known effects on health. The use of arsenic in numerous industrial processes has resulted in its presence in many biological and ecological systems, particularly aquatic ecosystems [70]. Arsenic occurs in several chemical forms in the aquatic environment. Arsenate (As^5+^) is usually the dominant form, but it can be biologically transformed to arsenite (As^3+^) and other chemical forms [70]. Algae are able to transform As^5+^ (Sanders, 1980), but are also targets of arsenic toxicity [71]. The toxicity of arsenic depends on its chemical form [72] and can be modified by phosphate. Arsenic was shown to be less toxic to phytoplankton communities [73]. Algae in arsenic contaminated environments develop resistance to arsenic toxicity and tend to accumulate and transform different chemical forms of arsenic [74]. The present investigation confirmed that As^5+^ is generally less toxic to the growth of *P. subcapitata*.

Lead concentrations below 0.320 mg L^−1^ were found to have little or no effect on the growth of free algal cells, but significantly stimulated the growth of immobilized algae. Further increases in Pb^2+^ concentration, however, caused significant growth inhibition. Pb was significantly more toxic to free (EC_50_ = 3.3 mg L^−1^) than to alginate immobilized (EC_50_ = 5.0 mg L^−1^) cells (*p* ≤ 0.05), with the growth of both cultures completely inhibited at Pb concentrations of 32.0 mg L^−1^ [41,75].

The physiological requirement of algae for Pb remains unclear. Lead has been regarded as a non-essential trace metal with no explicit positive effects or direct nutritional or biochemical functions in algae. However, certain species of algae have been reported to accumulate lead [3]. Lead has weakly toxic effects on photosynthesis, respiration, and cell division of various species of algae [76]. The results of the present study indicate that, similar to previous studies, Pb^2+^ had weak toxic effects on algal growth.

Although Sr^2+^ was the least toxic metal of those tested, it also did not stimulate the growth of either free or immobilized algal cells. Both cultures showed slight dose-dependent growth inhibition. The EC_50_ of Sr^2+^ was 160 mg L^−1^ for free cells and 180 mg L^−1^ for immobilized cells, with the growth of both cultures observed even at the highest concentration of Sr^2+^ tested (320 mg L^−1^).

Strontium constitutes about 0.02–0.03% of the Earth’s crust and is present in igneous rocks in amounts averaging 375 ppm [77]. As a result, that strontium is ubiquitous in the geosphere at relatively high concentrations, it is present in almost all living organisms including humans [78,79]. Growing concerns about the toxicity of Sr^2+^ to humans has led to investigations of its environmental fate and routes of exposure.

Aquatic environments contain appreciable amounts of strontium [79], and aquatic organisms, including algae, have been shown to accumulate strontium [80]. The test alga showed observable growth at surprisingly high Sr^2+^ concentrations (EC_50_ = 160 mg L^−1^ and EC_100_ > 320 mg L^−1^), a finding suggesting that *P. subcapitata* may be useful in regulating Sr^2+^ uptake. The tolerance of this species to high Sr^2+^ concentrations suggests that this species may retain and bioaccumulate sublethal concentrations of this heavy metal ion. Consumers of algae, including humans, may therefore be at risk if these algae flourish in Sr^2+^ contaminated aquatic ecosystems.

The main objective of the present study was to compare the sensitivity of free and alginate-immobilized algal cells to toxic effects of metal ions. Except for Cu^2+^ and Se^4+^, the EC_50_ values obtained with alginate-immobilized cultures were significantly (*p* ≤ 0.01) higher than those reported for free cell cultures. This finding indicates that the tested heavy metals are relatively less toxic to alginate-immobilized than to free algal cells.

These findings are in good agreement with those of previous studies. For example, calcium alginate beads were able to remove Hg^2+^ from aqueous solutions, with the removal being significantly greater with *Chlorella*-alginate beads [27,41,81]. These findings suggested that alginate matrices may protect *Chlorella emersonii* cells, enabling significant algal growth within alginate beads coupled with greater Hg^2+^ removal from solution. Hg^2+^ removal may be due to chelation by alginate, volatilization, and specific cellular metabolic activities. Immobilized algae may act as a biological ion exchanger to efficiently adsorb Hg^2+^ from contaminated ground water [13,27,41], and algae free calcium alginate matrices were found to adsorb Cu from aqueous solutions [82].

A study of the effects of chromium and nickel on the growth of and nitrogen fixation by free and alginate-immobilized cultures of the cyanobacterium *Aulosira fertilissima* found that cell immobilization could protect the organism against concentrations of both heavy metals that are lethal to free cells [82]. Cell immobilization also resulted in significant protection against sublethal concentrations of chromium and nickel.

The results of the present study suggest that immobilization protected *Pseudokirchneriella subcapitata* cells against heavy metal toxicity. The chemical properties of alginate may ameliorate the toxicity of these metal ions. Alginate, a mixture of polyguluronic acid and polymannuronic acid, has abundant hydroxyl groups that bind metal ions (e.g., Cd^2+^, Cu^2+^, Pb^2+^, Cr^3+^, and Hg^2+^), limiting their contact with cells and protecting against metal toxicity [18,75]. In addition to metal chelation, ion exchange can contribute to metal adsorption by alginate [20,27,62,83].

Alginate matrices were found to support relatively high growth and metabolic activities of immobilized algal cells, as well as efficiently and detoxifying metal ions. Algae utilize various mechanisms to regulate the uptake, biotransformation, and detoxification of metals, suggesting that the reduced toxicity of metals toward alginate matrices containing *P. subcapitata* cells results from the chelation and ion exchange properties of alginate in addition to the active metal detoxifying mechanisms of immobilized cells.

Algal cells immobilized in suitable matrices may be used to develop various algal biosensors to assess toxicity and environmental quality [15,16,75]. Caution should be exercised in using alginate immobilized cells to assess the toxicity of heavy metals, because alginate itself can ameliorate metal toxicity.

Free and immobilized *P. subcapitata* cells were observed to respond similarly to several pesticides, suggesting that batteries of immobilized algae can replace free cultures in assessing the toxicity of pesticides [17,84]. This finding was not observed, however, when testing the toxicity of heavy metal ions, with immobilized cells being less sensitive than free cells to these ions.

## 4. Conclusions

The present study tested the effects of 12 heavy metals that are widely distributed in aquatic environments on the growth of free and alginate-immobilized cultures of the standard test alga, *P. subcapitata*. Some of these metals were essential and others non-essential to algal growth. In general, free algal cells were more sensitive to metal toxicity than alginate-immobilized cells. Lower concentrations of some of these metals (e.g., Cu, Al) stimulated cell growth, whereas high concentrations of all metals sharply reduced the growth of both free and alginate-immobilized algal cells. Although aluminum is not considered a nutrient for microorganisms, the present study found that algal cell growth was significantly stimulated by 0.320 mgL^−1^ Al, suggesting the need to determine whether Al^3+^ is an essential micronutrient for this species of algae. The utility of the EC_50_ values generated in this study is not limited to comparisons of immobilized and free cells. The information derived from assays of free cells can be used to assess the environmental hazards of the tested metals. Except for Cu^2+^ and Se^4+^, the EC_50_ values of the 10 other metals were significantly higher for alginate-immobilized than for free cell cultures, indicating that these heavy metal ions were less toxic to alginate-immobilized algae and that alginate may play a crucial role in ameliorating metal toxicity. The chemical properties of alginate, including metal chelation and ion exchange activities of alginate, may account for the reduced toxicity of these metal ions to alginate-immobilized than to free algal cells. Toxicity determinations based solely on immobilized algal cells may therefore be inaccurate.

## Figures and Tables

**Table 1:** Chemical compounds tested.

Name	Formula	Tested as Metal Ions	Obtained from
Cadmium chloride	CdCl_2_·2½ H_2_O	Cd^2+^	BDH, Dubai, UAE
Cobalt chloride	CoCl_2_·6H_2_O	Co^2+^	MERCK, Darmstadt, Germany
Mercuric chloride	HgCl_2_	Hg^2+^	MERCK, Darmstadt, Germany
Cupric chloride	CuCl_2_·2H_2_O	Cu^2+^	MERCK, Darmstadt, Germany
Nickel sulfate	NiSO_4_·6H_2_O	Ni^2+^	MERCK, Darmstadt, Germany
Zinc chloride	ZnCl_2_	Zn^2+^	MERCK, Darmstadt, Germany
Chromium nitrate	Cr (NO_3_)_3_·9H_2_O	Cr^3+^	MERCK, Darmstadt, Germany
Aluminum chloride	AlCl_3_·6HO	Al^3+^	MERCK, Darmstadt, Germany
Selenium dioxide	SeO_2_	Se^4+^	MERCK, Darmstadt, Germany
Sodium selenite ^a^	Na_2_SeO_3_·5H_2_O	Se^4+^	MERCK, Darmstadt, Germany
Sodium arsenate	Na_2_HAsO_4_·7HO	As^5+^	MERCK, Darmstadt, Germany
Lead nitrate	Pb (NO_3_)_2_	Pb^2+^	BDH, Dubai, UAE
Strontium chloride	SrCl_2_·6H_2_O	Sr^2+^	MERCK, Darmstadt, Germany

^a^ This compound was used to test Se, although its toxicity may be due to selenite anion (Bringmann and Kuhn, 1980) Bringmann, G. and Kuhn, R. (1980): Comparison of the toxicity thresholds of water pollutants to bacteria, algae, and protozoa in the cell multiplication inhibition test. Water Res. 14, 231–241. (This is the references it needs a number).

**Table 2:** Toxicity of some heavy metals on growth of free and alginate-immobilized cells of the test alga *Pseudokirchneriella subcapitata*, toxicity is reported as mean values of EC_50_ and EC_100_ (n = 3).

	EC_50_ (mg L^−1^) ^a^				EC_100_ (mg L^−1^) ^b^		
	Free Cells		Immobilized Cells		Free Cells		Immobilized Cells
Chemical	Tested as	Old ^c^		New ^d^	Old		New
CdCl_2_·2½ H_2_O	Cd^2+^	0.018	0.09 ^***^^e^	0.09	0.1	1.0	1.0
CoCl_2_·6H_2_O	Co^2+^	0.03	0.06 ^***^	0.061	0.32	1.0	1.0
HgCl_2_	Hg^2+^	0.039	0.06 ^***^	0.063	0.032	1.0	1.0
CuCl_2_·2H2O	Cu^2+^	0.048	0.05	0.05	0.1	0.1	0.1
NiSO_4_·6H_2_O	Ni^2+^	0.055	0.03 ^***^	NT ^f^	0.1	3.2	NT
ZnCl_2_	Zn^2+^	0.08	0.1 ^*^	0.1	0.32	0.32	0.32
Cr(NO_3_)_3_·9H_2_O	Cr^3+^	0.2	0.3 ^***^	0.3	1.0	3.2	3.2
AlC_3_·6HO	Al^3+^	0.75	1.8 ^***^	NT	1.0	3.2	NT
SeO_2_	Se^4+^	1.2	1.4	NT	3.2	3.2	NT
Na_2_SeO_3_·5H_2_O	Se^4+^	3.0	3.0	NT	32	32	NT
Na_2_HAsO_4_·7HO	As^5+^	3.0	4.0 ^*^	4.0	32	32	32
Pb(NO_3_)_2_	Pb^2+^	3.3	5.0 ^*^	5.0	32	32	32
SrCl_2_·6H_2_O	Sr^2+^	160	180 ^***^	NT	>320	>320	NT

^a^ EC_50_ is the metal concentration inhibiting algal growth by 50% compared to control culture. Values were interpolated from dose–response curves. ^b^ EC_100_ is the metal concentration giving no observable algal growth. Values were visually inspected. ^c^ Beads were prepared, poured into flask containing AAM nutrient solution and left in dark at 4 °C. Beads were used after nine months of preparation. ^d^ Freshly prepared beads. ^e^ Differences between EC_50_ of free and immobilized cells are, * Significant (*p* < 0.05), *** Very highly significant (*p* < 0.001). ^f^ NT = not tested.
